# Possible Immunotherapeutic Strategies Based on Carcinogen-Dependent Subgroup Classification for Oral Cancer

**DOI:** 10.3389/fmolb.2021.717038

**Published:** 2021-08-23

**Authors:** Jiwei Sun, Qingming Tang, Junyuan Zhang, Guangjin Chen, Jinfeng Peng, Lili Chen

**Affiliations:** ^1^ Department of Stomatology, Union Hospital, Tongji Medical College, Huazhong University of Science and Technology, Wuhan, China; ^2^ School of Stomatology, Tongji Medical College, Huazhong University of Science and Technology, Wuhan, China; ^3^ Hubei Province Key Laboratory of Oral and Maxillofacial Development and Regeneration, Wuhan, China

**Keywords:** immunotherapies, oral squamous cell carcinoma, immune microenvironment, smoking, areca nut

## Abstract

The oral cavity serves as an open local organ of the human body, exposed to multiple external factors from the outside environment. Coincidentally, initiation and development of oral cancer are attributed to many external factors, such as smoking and drinking, to a great extent. This phenomenon was partly explained by the genetic abnormalities traditionally induced by carcinogens. However, more and more attention has been attracted to the influence of carcinogens on the local immune status. On the other hand, immune heterogeneity of cancer patients is a huge obstacle for enhancing the clinical efficacy of tumor immunotherapy. Thus, in this review, we try to summarize the current opinions about variant genetic changes and multiple immune alterations induced by different oral cancer carcinogens and discuss the prospects of targeted immunotherapeutic strategies based on specific immune abnormalities caused by different carcinogens, as a predictive way to improve clinical outcomes of immunotherapy-treated oral cancer patients.

## Introduction

Oral squamous cell carcinoma (OSCC) serves as one of the most important subtypes in head and neck squamous cell carcinoma (HNSCC), diagnosed cases of which have mounted to more than 600,000 worldwide, along with 50,000 new cases each year (Rahman et al., 2019). Unlike many other types of cancers whose pathogenesis is mainly explained by innate genetic alterations, OSCC is mainly related to some classical environmental risk factors such as tobacco and alcohol (Solomon et al., 2018). This phenomenon is easy to understand as the oral cavity is an open organ exposed to the outside environment and has broad interactions with environmental factors. On the other hand, with the development of oncogenic studies, the role of abnormality in the tumor microenvironment has been identified to be more and more important (Binnewies et al., 2018). Recently, many types of immune cells, such as M2 macrophages, regulatory T (Treg) cells, and myeloid-derived suppressor cells (MDSCs), were discovered to exert a pro-tumor influence on oral carcinogenesis (Dar et al., 2020; Li et al., 2020). The cell–cell communications mediated by extracellular vesicles have been identified as crucial mechanisms contributing to tumor progression in many types of carcinomas. Similarly, extracellular vesicles from multiple origins could get involved in many tumor-associated processes, including proliferation, metastasis, and drug resistance during oral cancer development (Xie et al., 2019). The above clues together indicated whether environmental risk factors could promote OSCC progress *via* deregulation of the tumor microenvironment. In the perspective of tumor therapy, immunotherapies, including check-point therapy and molecule-targeted strategy, have been making significant advances and improving the prognostic outcome of tumor patients to a great extent (Kennedy and Salama, 2020). However, immunotherapy has failed to be the prior strategy for OSCC treatment, partly due to the heterogeneity of OSCC patients, as multiple types of risk factors, for example, smoking and drinking, were involved in the initiation and progression of OSCC. Considering the fact that the huge financial burden and surgical complications are the main blockades for favorable clinical outcomes of OSCC patients, a therapeutic strategy based on patient-specific risk factors is extremely necessary to direct the application of different types of immunotherapies to different patients. This kind of strategy might overcome the heterogeneity of OSCC patients and realize individual-based diagnosis and therapy for OSCC patients, paving the way for the future of OSCC immune therapies.

## Possible Carcinogens for the Development of OSCC

Exogenous carcinogen-induced tumorigenesis acts as a significant feature of OSCC, distinguishing it from other cancers. Until now, multiple kinds of substances were regarded as possible OSCC carcinogens.

Among all the possible carcinogens for OSCC, cigarette, alcohol, and areca nuts were the most prevalent and well-acknowledged carcinogens (Kumar et al., 2016). Lots of clinical and epidemiological research has identified the strong relationship between smoking and OSCC. Results of a study based on 1,114 participants showed that the risk of OSCC among non-drinkers amounted to the quantity of smoking (Blot et al., 1988). In different regions, such as East Asia, Iran, and Brazil, OSCC patients all exhibited a high percentage of smoking habits, indicating a general influence of smoking on OSCC initiation (Razavi et al., 2015; Bezerra et al., 2018; Hashibe et al., 2019). Furthermore, OSCC patients with a smoking habit showed more aggressive disease features and poorer prognostic outcomes than non-smoking OSCC patients, indicating that smoking might also contribute to the progression and aggression of OSCC (Al Feghali et al., 2019). Alcohol abuse has been implicated as a high risk factor in many types of cancers, including OSCC (Ng et al., 1993), esophageal cancer (Castellsagué et al., 1999), larynx cancer (Bosetti et al., 2002), colorectal cancer (Cho et al., 2004), and pancreatic cancer (Korc et al., 2017). For OSCC, alcoholic beverages have been implicated as an important carcinogen in the etiology of oral cancer since the 1980s (Kabat and Wynder, 1989). Risk of OSCC among non-smokers was also confirmed to increase along with alcohol consumption (Blot et al., 1988). Specifically, a combination of alcohol abuse and smoking could enhance the carcinogenic effect of each other, suggesting a synergistic effect of alcoholism during OSCC development (Castellsagué et al., 2004). It is well known that the habit of chewing areca nuts is widely popular in Southeast Asia, and its positive role in the development of oral precancerous lesions and OSCC has been fully accepted as well (Li et al., 2016). As the areca nut industry was growing fast worldwide, more and more public and medical sources were paid due to betel chewing–induced OSCC (Hu et al., 2017).

Besides the above carcinogens, periodontal infection was also reported to be associated with OSCC development. Lower frequency of tooth-brushing and fewer dental visits, which were highly related with periodontal infection, were all associated with OSCC development. The poorer overall survival of OSCC patients with poor periodontal hygiene further suggested possible roles of periodontal infection in OSCC. With the development of society and subsequent changes in traditional concepts, the frequency of oral sexual behavior has mounted to a high level, especially in young adults (Holway and Hernandez, 2018). This behavior shift makes the oral cavity exposed to a totally new environment. Clinical trials have revealed that changes in sexual behaviors trend toward a higher incidence of oral human papillomavirus (HPV) infection (Chaturvedi et al., 2015). This phenomenon just coincides with the conclusion that the percentage of HPV-positive oropharyngeal carcinomas has risen from 16.3% in the 1980s to 72.7% in the 2000s (Chow, 2020). Obviously, oral sex–mediated HPV exposure has become a newly emerging risk factor for oral and pharyngeal carcinomas.

In addition, some novel perspectives about OSCC-related external carcinogens have been implicated. The presence of some unhealthy components inside the oral cavity, including residue dental roots and crowns, as well as improper dental prothesis, was identified to promote the malignant transformation of the normal oral epithelium due to its persistent physical stimulus. Besides, global nutrition deficiency might also be related to OSCC. Deficiency of vitamin A was identified to have a correlation with the occurrence of oral leukoplakia, a type of oral precancerous lesion (Sankaranarayanan et al., 1997). Loss of vitamin D might also act as a contributor to OSCC progression (Verma et al., 2020). Persistent intake of hot water and food is an acknowledged risk factor for esophagus carcinoma, and this stimulus, along with other stimulatory factors including pungent passing through the oral cavity, might also promote the formation of malignant oral lesions. In some special regions of the world, for example, New Zealand, where strong illumination exists throughout the year, UV radiation was also considered a possible carcinogen for skin carcinoma and OSCC (Yakin et al., 2017).

## Abnormality of Genome Landscape in OSCC

Traditional concepts claimed that initiation and development of oral cancer are due to a sum of self- or risk factor–induced genetic changes that would lead to alterations in the activation of oncogenes and inactivation of tumor suppressor genes (Pérez-Sayáns et al., 2009; Irimie et al., 2016). Multiple types of genes controlling cell proliferation, DNA repair, angiogenesis, and other pro-tumor biological processes have manifested significant variations between OSCC and normal oral tissues.

## Abnormality of Immune Microenvironment in OSCC

The current hot spot in cancer oncology research, apart from genetic variations, mainly lies in the tumor microenvironment, which contributes a lot to local tumorigenesis by promoting cellular proliferation, metabolism, metastasis, and so on (Casey et al., 2015). In the perspective of OSCC, although the heterogeneity of immune cell infiltration in the OSCC microenvironment makes it difficult to describe the total immunological landscape, more and more studies have been dedicated to figuring out this challenge.

### Abnormal Production of Cytokines

**FIGURE 1:**
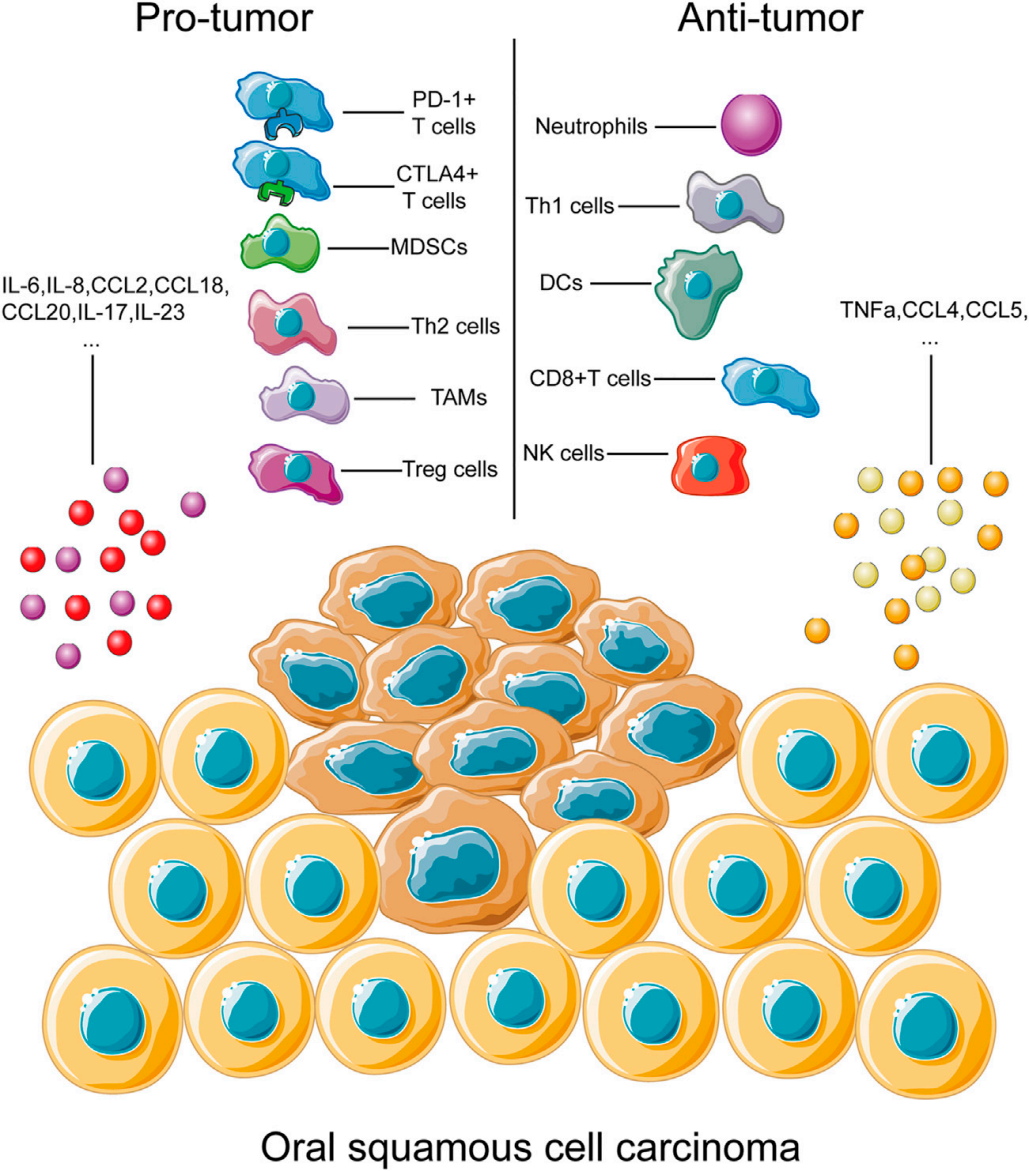
Immune cells and cytokines associated with OSCC progression. This figure depicts the main types of immune cells and cytokines associated with progression of OSCC development. During OSCC progression, immune cells and cytokines could be divided into two groups, the pro-tumor part and the anti-tumor part. Immune cells, including PD-1^+^ and CTLA4^+^ T cells and TAMs, have been identified to play roles in tumor progression through multiple pathways, as discussed above. Similarly, immune cells contributing to the anti-tumor process, Th1 cells and NK cells, for example, were also listed inside. A complete overview of immune cells involved in tumor development would help us better understand the complexity of immune regulation of OSCC.

Abnormal expression of chemokines plays an important part in the immunomodulation of the tumor microenvironment as recruitment and activation of immune cell subtypes are largely mediated by the interaction between chemokines and chemokine receptors (Nagarsheth et al., 2017). The chemokine-mediated regulating network exhibited its complexity *via* bidirectional pro-tumor and anti-tumor functions (Figure 1).

IL-2 functions as an important growth factor for T cell subpopulations, and activation of CD4^+^ T cell differentiation by IL-2 has been verified. In addition, effector and memory CD8^+^ T cell responses could also be induced by IL-2 (Spolski et al., 2018), indicating its crucial role in the T cell–mediated anti-tumor process.

A large amount of evidence indicated crucial roles of IL-10 in the anti-tumor process. CD8^+^ T cell function and memory could be ignited by IL-10 exposure (Foulds et al., 2006). Elevation of granzyme B and activation and interferon-γ (IFN-γ) expression, along with subsequent CTL infiltration, have also been identified (Emmerich et al., 2012). Direct stimulation of NK cells by IL-10 would contribute to its anti-tumor effects (Lauw et al., 2000), while IL-10 could also indirectly mediate NK cell activation *via* inhibition of ROS secretion by TAMs (Mocellin et al., 2004).

IL-35 is another commonly acknowledged regulator in the tumor microenvironment. As a member of the IL-12 family, its immune suppressive role would render a pro-tumor status. Pro-tumor neutrophils induced by IL-35 could result in malignant progression of local tumor tissues (Zou et al., 2017). In addition, Tregs accumulated in tumor sites are the main resource of IL-35, and anti-IL35 treatment showed a similar effect to that of the depletion of Tregs. Inhibition of Th17 cell growth and function by IL-35 were also discovered (Niedbala et al., 2007), indicating that IL-35 might inhibit anti-tumor effects partially through a Th17 blockade.

IL-6 is a crucial chronic inflammatory mediator, higher levels of which have been observed in multiple types of cancers. Several major pro-tumor activities, including growth, invasion, and angiogenesis, have been identified to be closely correlated with IL-6 overexpression. In addition, the blockade of type 1 immune response (Tsukamoto et al., 2018), elevation of Treg cells (Kato et al., 2018), expansion of MDSCs, and activation of stromal fibroblasts could also be induced by IL-6, thus contributing to tumor development (Hanazawa et al., 2018).

IL-8 normally acts as a kind of chemokine, recruiting the accumulation of leukocytes (Alfaro et al., 2017). While in the tumor microenvironment, the high affinity of IL-8 to CXCR1 and CXCR2, the activation of which has been demonstrated to play an important role in tumor progression (Campbell et al., 2013; David et al., 2016), would contribute a great deal to the malignant process. Besides, an IL-8–induced increase in MDSC recruitment would accelerate the chronic inflammatory status of local tumor sites (Chi et al., 2014).

Similarly, IL-17 also played pro-tumor roles during tumor progression. Chronic exposure of IL-17 would lead to a pro-tumor microenvironment *via* production of inflammatory mediators, mobilizing myeloid cells and a phenotypic switch of stromal cells (Zhao et al., 2020).

TNF-α is a key pro-inflammatory cytokine, which exhibited a dual function in tumor progression. Stromal cells and cancer cells could both be sources of TNF-α. On the one hand, high levels of cytotoxic potential from TNF-α could render destruction of tumor vasculature, necrosis and apoptosis of cancer cells, and facilitation of drug accumulation inside tumor sites. On the other hand, studies also showed that TNF-α secreted by host cells surrounding tumor tissues could instead construct an inflammatory status and promote tumor progression (Egberts et al., 2008; Sethi et al., 2008).

As for OSCC, differential production of some types of chemokines is significantly associated with carcinogenesis.

When it comes to chemokines, accumulations of CCL20, CCL18, CCL4, and CCL2 were identified to promote tumor progression in OSCC (Li et al., 2014; Lee et al., 2017; Wang et al., 2017; Lien et al., 2018). Meanwhile, gene polymorphisms of CCL4 and CCL5 were highly associated with OSCC susceptibility (Weng et al., 2010; Lien et al., 2017). These deregulated chemokines have been shown to play roles in microenvironmental immunomodulation *via* recruitment of Treg cells, macrophages, MDSCs, and so on (Nagarsheth et al., 2017). As for inflammatory cytokines, interleukin (IL) 6, IL-8, and tumor necrosis factor-alpha (TNF-α) have been identified in terms of their potential roles as diagnostic biomarkers for OSCC (Sahibzada et al., 2017). Association between the gene polymorphism of IL-2 and OSCC has also been discovered (Singh et al., 2017). IL-23 could contribute to the progression of premalignant oral lesions to OSCC (Caughron et al., 2018), while IL-17 was significantly linked to the overall survival status of HNSCC (Lee et al., 2018). It is widely acknowledged that complex interacting networks of inflammatory cytokines could control recruitment, activation, and suppression of immune cells. Thus, the discussed abnormality of inflammatory cytokines in OSCC might result in a pro-tumor immune landscape in OSCC.

### Abnormal Tumor-Infiltrating Immune Cells

Tumor-infiltrating cells are those immune cells located in the local tumor microenvironment, which have been identified to play crucial roles in either pro-tumorigenesis or anti-tumorigenesis and have significant prognostic value in cancer development (Shang et al., 2015). Immune cells inside the tumor microenvironment could commonly be divided into two groups, the myeloid cell subgroup and the lymphocyte subgroup, all of which work together to form a comprehensive and interactive immune regulating network to influence the complexity of the tumor immune microenvironment.

#### Tumor-Related Myeloid Cells

Traditionally, myeloid cells acted as major components for host protection. They have changed evolutionally as barriers against variant infections and contributors to tissue remolding. However, during tumor development, myeloid cells would play complicated roles (Gabrilovich, 2017).

##### Myeloid-Derived Suppressor Cells

Myeloid cells which are CD11b- and Gr-1–positive and exhibited a strong immune suppressive effect have now been defined as myeloid-derived suppressor cells (MDSCs). Main subtypes of MDSCs are polymorphonuclear (PMN-MDSCs) and mononuclear (M-MDSCs). Although the suppressive effect of MDSCs could cover multiple types of immune cells, T cells are their main targets (Gabrilovich et al., 2012). Production of NO and variant cytokines induced by M-MDSCs could efficiently suppress T cell activity, as a NO-associated T cell receptor blockade would reduce the antigen presentation process (Koehn et al., 2015), while formation of antigen-specific T cell tolerance is a main mechanism for PMN-MDSCs (Gabrilovich et al., 2012). Besides, production of reactive oxygen species (ROS) is also essential for this process. On the other hand, MDSCs could also participate in the remolding process of the tumor microenvironment and tumor angiogenesis *via* VEGF, bFGF, and MMP9 (Casella et al., 2003; Shojaei et al., 2009).

##### Neutrophils

Neutrophils are the first line against multiple infections of the host. However, their plasticity in the tumor microenvironment puts them into both pro-tumor and anti-tumor roles (Giese et al., 2019). Generation of ROS, reactive nitrogen species (RNS), and hydrogen peroxide could direct cancer cell–specific death, which is the main mechanism for neutrophil-induced anti-tumor roles (Granot et al., 2011), while in addition to their cytotoxic effect, abnormal production of ROS and RNS would result in oxidative DNA damage and genetic variations (Güngör et al., 2010). It is also well known that neutrophil-extracellular traps (NETs) generated in the tumor microenvironment would result in migration and invasion of cancer cells (Park et al., 2016). Secretion of MMPs from neutrophil granules might promote malignant development *via* migration, proliferation, and angiogenesis (Ardi et al., 2007; Das et al., 2017).

##### Dentritic Cells

Dendritic cells (DCs) are commonly regarded as the activator of T cells *via* transporting cancer-associated antigens. Initiation, polarization, and direction of T cells in the tumor microenvironment as well as recycling lymph nodes by DCs are the main mechanisms for DC-induced tumor suppression, during which the CD8^+^T cell is their main target (Gardner et al., 2016). However, suppression of DCs in the tumor microenvironment would block this process, rendering a non-immunogenic DC phenotype switch. Thus, stimulatory and suppressive signals in the tumor microenvironment aiming at DCs, cytokines, and cell–cell communication, for example, would regulate DC-related T cell immunogenic functions.

##### Tumor-Associated Macrophages

Tumor-associated macrophage (TAM) is a subtype of infiltrating macrophage contributing to local tumor growth, metastasis, and neovascularization (Zhu et al., 2017). Infiltrating TAMs in OSCC is also associated with tumorigenesis. CD163, a common marker for TAMs, was observed to be elevated in OSCC tissues (Stasikowska-Kanicka et al., 2018a), suggesting its possible relationship with oral carcinogenesis. Coincidentally, the same phenomenon was observed in oral precancerous lesions (Boas et al., 2013). Besides, CD204, another TAM marker, was shown to be linked to the progress from oral premalignant lesions to OSCC (Kouketsu et al., 2019). Using a xenograft model, irradiation-induced M2 macrophage accumulation showed the potential to promote oral tumor recurrence *via* enhancement of neovascularization (Okubo et al., 2016). The *in vitro* experiment remodeling the tumor environment confirmed the mutual promoting effect between oral cancer cells and TAMs (Essa et al., 2016), and the Gas6/Axl signaling pathway was further confirmed to enhance the epithelial-mesenchymal-transition of oral cancer cells (Lee et al., 2014). Apart from M2 macrophages, M1 subtype TAMs also played a positive role in OSCC (Xiao et al., 2018). In the early tumor stage, local resident macrophages act in cooperation with other innate immune cells to initiate inflammatory responses to reduce tumor progression, through some direct effects, for example, ROS generation, and some indirect pathways, such as regulation of Th1 responses (Joyce and Pollard, 2009; Murray and Wynn, 2011). It could be concluded from the above that TAM is significantly involved in the pathogenesis of OSCC.

#### Tumor-Related Lymphocytes

##### CD8^+^T Cell

The CD8^+^T cell is a generally recognized anti-tumor defender of the host and serves as one of the most crucial effector cells in anti-cancer immunity, dysfunction of which would result in a severe barrier for cancer elimination (He et al., 2019). Loss of CD8^+^T cells has contributed to tumorigenesis in many types of cancers. In OSCC, the CD8^+^T cell was shown to decrease in either OSCC tissues or precancerous lesions (Stasikowska-Kanicka et al., 2018a). High CD8^+^T cell percentage could also predict a better overall survival and disease-specific survival rate in OSCC (Shimizu et al., 2019). Immunological staining further revealed an increase in CD8^+^T cells in OSCC with better prognosis (Stasikowska-Kanicka et al., 2018b). The expression level of PD-L1, an immune checkpoint blockade targeting cytotoxic T cells, was highly unregulated in OSCC (Stasikowska-Kanicka et al., 2018a; Stasikowska-Kanicka et al., 2018b), indicating a loss-of-function status of T cells in OSCC. In the translational medical perspective, the anti-tumor effect of radiotherapy in OSCC was also verified to be partly attributed to the activation of CD8^+^ T cells (Suwa et al., 2006). These clinical experimental results together come to the conclusion that CD8^+^ T cells play an anti-tumor role in OSCC, the abnormality of which would help in tumorigenesis.

##### CD4^+^T Cells

T cells expressing CD4 glycoprotein are another crucial T cell subtype called the CD4^+^T cell, and their functions in the tumor microenvironment are extremely complicated, due to multiple subgroups of CD4^+^T cells, including Th (T helper)1 cells, Th2 cells, Th9, Th17, Th22, and T regular cells (Treg).

Th1 cells show some anti-tumor effects mainly *via* their large amount of IFN-γ production, along with some chemokines to recruit and prime effector CD8^+^T cells. Also, NK cells and M1 macrophages could be recruited and activated by Th1 cells in local tumor sites for tumor elimination (Nishimura et al., 1999). By targeting of tumor stroma and subsequent angiogenesis blockade, tumor growth could be inhibited in an IFN-γ–mediated way by CD4^+^T cells (Qin and Blankenstein, 2000).

Th2 cells have been verified to play some contradictory roles in tumor progression. Secretion of IL-4 by Th2 cells would mediate transport of macrophages and eosinophils into tumor sites for anti-tumor actions (Tepper et al., 1989), and this immune transfer function is the main mechanism for the Th2-mediated anti-tumor effect. On the other hand, antigen-specific effector Th2 cells have been reported to promote cancer development, and IL-5 secreted by them might be the reason behind this pro-tumor effect (Tatsumi et al., 2002).

Th17 cells are an important subgroup participating in the anti-infection process. A pro-inflammatory microenvironment induced by secreted IL-17a and IL-23 would promote tumor progression *via* elevating angiogenesis and inhibiting infiltration of CD8^+^T cells. Only a small amount of Th17-associated cytokine exposure could remarkably facilitate cancer progression (Lee et al., 2012), while some studies support that a high level of IL-17 would result in an anti-tumor immune status (Numasaki et al., 2005). This contradictory phenomenon suggested that the role of Th17 in tumor progression largely depends on the host status and local context.

Treg is a negative regulator in the adaptive immune system, suppressing the activation of immune responses and maintaining the immune balance (Sakaguchi, 2004). During tumor progress, excessive upregulation and activation of Treg cells would result in immune deficiency and subsequent immune escape of tumor cells, thus facilitating the development of tumorigenesis (Smigiel et al., 2014). In OSCC patients, elevated levels of Treg cell–associated cytokines were observed in peripheral blood (Gaur et al., 2014), while a higher frequency of Treg cells was also discovered in OSCC samples (Schwarz et al., 2008). Animal experiments using mice and dogs further verified an increase in Treg cells in OSCC (Horiuchi et al., 2010; De Costa et al., 2012). In tongue squamous cell carcinoma, a higher level of expression of Treg cells was significantly associated with a poorer survival rate, and accumulation of Treg cells was used to predict bad prognosis of patients (Hanakawa et al., 2014). Compared with healthy donors, levels of circulating Treg cells were also much higher in OSCC patients, along with a higher level of TGF-β, a Treg-associated cytokine (Lim et al., 2014). The above evidence together confirmed the pro-tumorigenesis value of Treg cells in OSCC.

##### Natural Killer Cells

The anti-tumor immunity of natural killer (NK) cells has long been regarded as a predominant effector against metastasis or hematological cancers, and more and more efforts have been applied to fully understand properties of NK cells (Guillerey et al., 2016). Escape of NK cell immune surveillance in OSCC tissues and inactivation of the NK cell status in peripheral circulation of OSCC patients has been recorded in clinical research (Dutta et al., 2015). Similarly, the downregulated NK cell status was also observed to be linked to higher invasive oral tumor areas (Türkseven and Oygür, 2010). A newly published meta-analysis indicated the possibility of the NK cell marker being a prognostic marker, considering the negative correlation between NK cell markers and the OSCC patient survival rate (Huang et al., 2019). The successful curative effect of NK cell immunotherapy in OSCC identified in an *in vivo* model further confirmed the crucial anti-tumor role of NK cells in OSCC (Greene et al., 2020).

## OSCC Carcinogen-Induced Immune Abnormalities

As predominant contributors to the progress of OSCC, multiple OSCC-related carcinogens, including smoking, drinking alcohol, chewing areca nuts, periodontal infection, and oral sexual behavior, have all been proven to be related to local immune abnormality to a great extent. A complete understanding of the immune status induced by carcinogens would help in possible recognition of the immune landscape of carcinogen-induced OSCC.

### Cigarette

**FIGURE 2:**
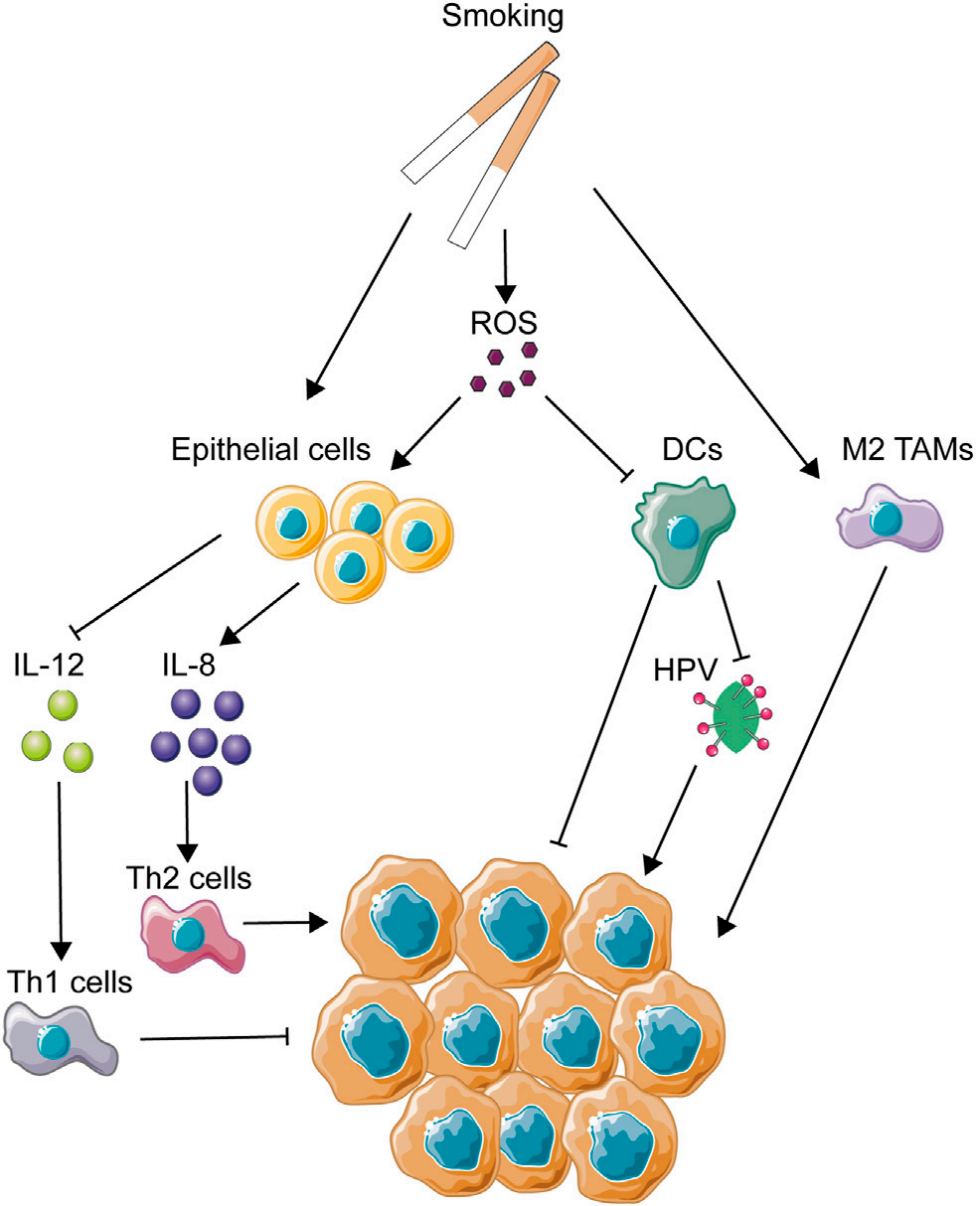
Mechanisms of smoking-related immune regulation in OSCC. This figure depicts several main immune-regulating activities associated with smoking. As one of the most important OSCC carcinogens, smoking could directly or indirectly regulate immune activities through activation of epithelial cells and immune cells and the production of ROS.

Cigarettes are a well-known risk factor for many oral diseases, including periodontitis (Kinane et al., 2017), halitosis (Jiun et al., 2015), oral leukoplakia (Granero Fernandez and Lopez-Jornet, 2017), and OSCC (Blot et al., 1988). Previous studies about the effect of smoking on carcinogenesis mainly focused on aberrant genetic alterations brought on by harmful compounds inside cigarettes. Accumulation of DNA adducts and oxidative DNA damage induced by tobacco smoking have been identified for a while (Phillips, 2002), and the subsequent genetic mutational signatures, such as TP53, P73, and MDM2 (Misra et al., 2009), were listed clearly, using sequencing methods (Alexandrov et al., 2016). However, little attention was cast onto the influence of microenvironmental changes caused by tobacco. The oral cavity is a local microenvironment whose stability would be extremely changed due to tobacco smoking (Figure 2). Thousands of reactive oxygen species (ROS) are generated in burning cigarettes (Huang et al., 2005), and ROS-attacked epithelial cells and cancer cells in the oral cavity would secrete lots of inflammatory mediators, thus leading to imbalance of host immunity in the oral cavity. It is evidenced that cigarette smoke could result in upregulation of IL-8 (Barnes, 2016) and downregulation of IL-12 by the oral epithelium (Vassallo et al., 2005a). As IL-12 is a main inducer of the Th1 response (Trinchieri, 2003), the phenomenon coincides with the observation that cigarette smoking would result in suppression of Th1 responses and generation of Th2 inflammatory reaction (Cozen et al., 2004), and excessive Th2 response would break the balance between Th1 and Th2. As has been known, Th2 immune polarization might result in some unexpected effects in carcinogenesis. CCL5-mediated recruitment and differentiation of Th2 cells enhanced the primary tumor burden and pulmonary metastases of luminal breast cancer (Zhang et al., 2015). NLRP3-activated polarization of Th2 cells also had a tumor-promoting impact on pancreatic cancer (Daley et al., 2017), further identifying the immunosuppressive role of Th2 in carcinogenesis. On the other hand, patients with higher accumulation of Th1 exhibited better and prolonged survival (Tosolini et al., 2011), precise evidence for the anti-tumor effect of Th1. Furthermore, some chemotherapies targeted at the tumor microenvironment also aim to enhance Th1 cytokine levels as an anti-tumor pathway (Berlato et al., 2017). Thus, smoking-induced imbalance of Th1/Th2 might be speculated to play a role in development of OSCC from an immunological perspective. Besides, IL-8 could act as a pro-tumorigenesis cytokine with its promotion of tumor proliferation, migration, and maintaining of stemness (Chen et al., 2014; Huang et al., 2015; Ding et al., 2017). As reported, the release of IL-8 induced by cigarette smoke (CS) was mainly from macrophages (Facchinetti et al., 2007) and airway epithelial cells (Mio et al., 1997). Interestingly, several research studies have reported that IL-8 was induced in oral squamous cell carcinoma cells (Tsunoda et al., 2016) and gingival epithelial cells (Mahanonda et al., 2009). Thus, upregulation of IL-8 by CS might play an important role in CS-induced tumorigenesis.

CS could also impose a great influence on immunological functions of dendritic cells. It has been reported that some components of CS, such as ROS, nicotine, and other chemicals inside, were involved in the influence on DCs (dendric cells), causing the suppression of DC-induced T cell activation and proliferation (Vassallo et al., 2005b; Kroening et al., 2008; Vassallo et al., 2008). DCs are considered to be main activators for both innate immunity and adaptive immunity, being highly efficient in generating fast and fierce immunological responses (Constantino et al., 2017). However, under the immunosuppressive influence of the tumor microenvironment, DCs always show a biological dysfunction in the cancerous background, as a way to help tumor evasion (Tang et al., 2017). Due to its central role in the initial phase of immunity activation, DC-based immunotherapy has been used in clinical trials since the mid-1990s and has been applied in many types of cancers such as melanoma, prostate cancer, malignant glioma, and renal cell carcinoma (Anguille et al., 2014). Thus, CS-induced dysfunction of DCs is considered as a contributor to the malignant development of the tumor microenvironment. In addition, CS extract has also been found to suppress production of antiviral cytokines from DCs (Mortaz et al., 2009). In nasopharyngeal carcinoma, CS extract has been proved to promote the infection of the Epstein–Barr virus (EBV), the enhancement of which is closely associated with the malignant development of nasopharyngeal carcinoma (Huang et al., 2017). The proportion of HPV-related OSCC has increased in the past 30 years in a longitude clinical survey in America (Chaturvedi et al., 2008), emphasizing the importance of HPV infection in OSCC development. Thus, it could be speculated that the decrease in antiviral capacity caused by CS might promote the colonization and replication of HPV in the oral cavity.

Smoking was found to increase the aggregation of alveolar macrophages but impair the normal functions of macrophages (Kotani et al., 2000; Hodge et al., 2003). The same phenomenon was identified *in vitro* (Kirkham et al., 2004). As is known to all, dysfunction of macrophages might help to promote the development of tumors. In addition, smoking could induce the polarization of M2 macrophages in alveoli (Bazzan et al., 2017). In an *in vivo* mouse model, smoking was identified to induce the polarization of tumor-associated macrophages and promote the development of pancreatic cancer in this way (Kumar et al., 2015). Thus, the dysfunction and M2 polarization of macrophages caused by smoking might partly explain the malignant transformation of the tumor microenvironment, leading to OSCC development as a result.

On the other hand, CS is a crucial modulator of host response to pathogens (Nuorti et al., 2000). Smokers are shown to be more likely to get infection of *Streptococcus pneumoniae* and *Tuberculosis* (Padrao et al., 2018). This feasibility of pathogen colonization might suggest a dysfunction of host immunity and destruction of microbial balance, leading to a low resistance to extraneous pathogens. Recently, the relationship between oral microbial dysbiosis and tumor development has been a hot topic, and a lot of evidence has revealed microbial dysbiosis as a contributor to carcinogenesis. Smoking was strongly identified to be involved in oral microbial variations through some clinical trials with large amounts of samples (Wu et al., 2016; Yu et al., 2017). Microbial diversity was decreased in smokers, and there were a reduction of phylum Proteobacteria and genera *Capnocytophaga*, *Peptostreptococcus*, and *Leptotrichia* and enhancement of *Atopobium* and *Streptococcus*. These abnormal changes of host microbial composition caused by smoking might be one of the reasons for the CS-induced effect of carcinogenesis.

### Alcohol

**FIGURE 3:**
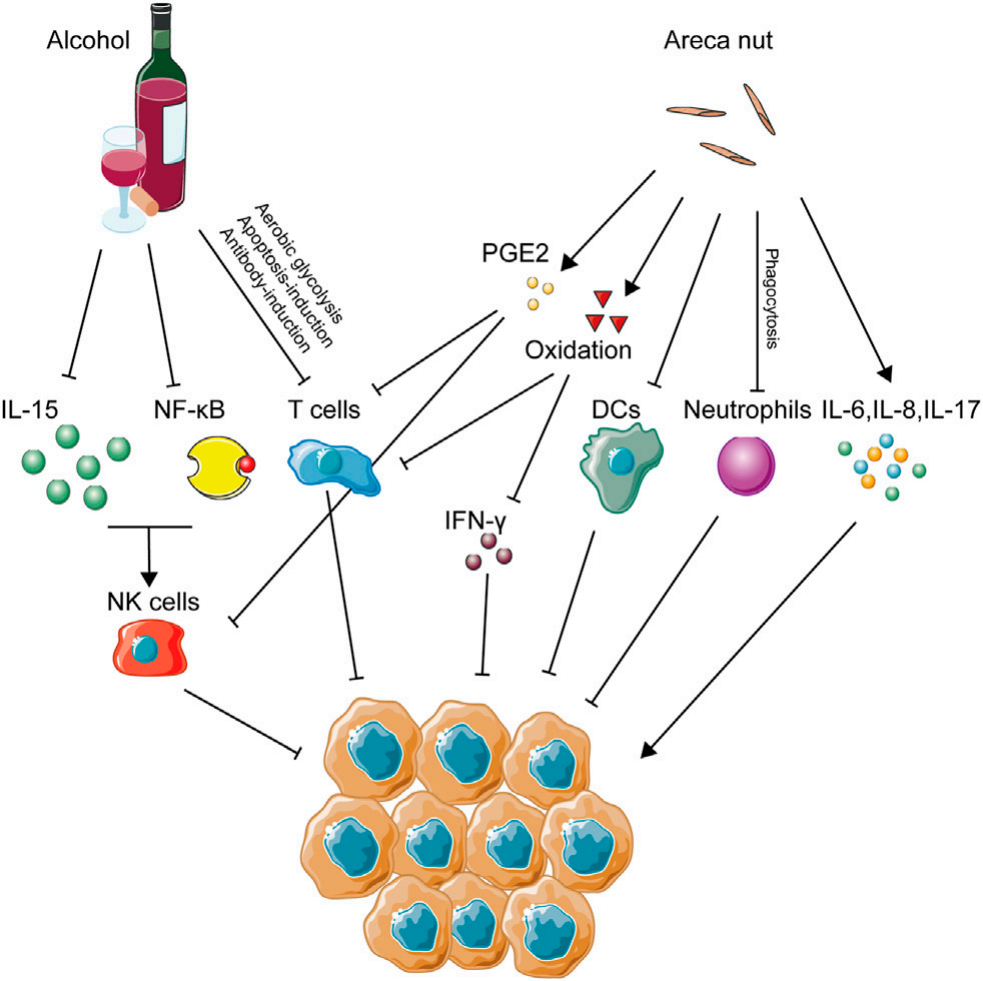
Immune activities caused by alcohol and areca nut consumption in OSCC. This figure depicts the immune-regulating networks induced by alcohol and areca nuts during OSCC development according to related studies. Specifically, inhibition of NK cells, T cells, DCs, and neutrophils, several main anti-tumor immune cells, was a remarkable feature of immune abnormalities induced by alcohol and areca nuts, indicating that immune inhibition might be excessively crucial in OSCC associated with these two carcinogens.

Alcohol abuse has been implicated as a high risk factor in many types of cancers, including OSCC (Ng et al., 1993), esophageal cancer (Castellsagué et al., 1999), larynx cancer (Bosetti et al., 2002), colorectal cancer (Cho et al., 2004), and pancreatic cancer (Korc et al., 2017). Alcohol dehydrogenase (ADH) and aldehyde dehydrogenase (ALDH) play crucial roles in the regular conversion of ethanol to acetate. When it comes to carcinogenesis, traditional opinions about alcohol-induced carcinogenesis support the abnormal metabolism of ethanol caused by variations of ADH- and ALDH-encoding genes serving as main contributors (Jelski et al., 2009). For example, this abnormality of ethanol metabolism could lead to increased generation of ROS from epithelial cells, which then activates cellular pathways, such as the nuclear factor κB (NF-κB) pathway and the mitogen-activated protein kinase (MAPK) pathway (Wu, 2006; Morgan and Liu, 2011), causing the malignant transformation of tumor cells. In addition, the RNS level of the epithelium was also elevated by alcohol stimulation. Accumulation of ROS and RNS would indirectly modify the immune microenvironment *via* its suppressive effect on T cells, NK cells, and macrophages (Figure 3). Besides this direct carcinogenic effect of alcohol, the function of host immunity is also under significant burden due to alcohol intake, which might partly explain the carcinogenesis of alcohol.

Alcohol intake, either acute or chronic, poses a great burden on NK cells. Alcohol abuse has long been regarded as a promoter for the development of hepatic diseases, such as hepatitis viral infection and liver fibrosis. It is reported that chronic ethanol consumption would accelerate virus-induced hepatitis through suppression of NK cell accumulation and cytotoxicity ability (Pan et al., 2006). Similarly, ADH3, a crucial enzyme in the metabolic process of alcohol, has great correlation with the development of hepatic fibrosis due to its suppression of NK cells (Yi et al., 2014). Abrogation of the antifibrotic effect of NK cells caused by alcohol was seen to increase the severity of alcoholic liver fibrosis (Jeong et al., 2008). In alcoholic hepatitis, a decreased frequency and reduction of the degranulation capacity of NK cells were also observed compared with healthy ones (Støy et al., 2015). When it comes to cancer development, alcohol abuse could also explain its carcinogenesis through NK cell variation, as acute alcohol ingestion has been demonstrated to cause a marked reduction of NK cell activity and, in this way, promote the tumor metastasis *in vivo* (Ben-Eliyahu et al., 1996). Metastasis of colon cancer cells into the liver was also increased by treatment of chronic alcohol consumption in a preclinical model (Im et al., 2016). In perspective of the count variation of NK cells, alcohol consumption has been identified to decrease the number of NK cells in the spleen (Blank et al., 1991) and peripheral lymph nodes (Zhang et al., 2011). Furthermore, the balance of thymus-derived and bone marrow–derived NK cells was also destroyed by alcohol intake (Zhang and Meadows, 2008). Besides, cytotoxicity and cell activity of NK cells was downregulated by treatment of alcohol (Wu et al., 1994), and enzymatic activity of granzyme A and B expressed by NK cells was suppressed, resulting in the loss of cell viability of NK cells (Spitzer and Meadows, 1999). On the other hand, some research focused on the explanations for alcohol-induced suppression of NK cells. Alcohol consumption could render a variation of the autonomic nervous system and reduction of pro-inflammatory cytokines from neuroendocrine and immune cells, leading to suppression of NK cell cytolytic activity (Boyadjieva et al., 2006; Chen et al., 2006). Downregulation of IL15 induced by alcohol consumption seemed like a way to suppress the availability of NK cells (Zhang et al., 2017), and this observation has been confirmed by a rescue experiment (Zhang and Meadows, 2009). Moreover, activity of the NF-κB pathway in NK cells, a crucial pathway for immune activation, was also suppressed by alcohol treatment (Zhou and Meadows, 2003).

T cell function is also under the influence of alcohol drinking. Alcohol-derived acetaldehyde has been proved to pose severe toxicity to the immune system, and recent study has confirmed its role in the downregulation of T cell function *via* inhibiting aerobic glycolysis and hampering the energy source of T cells (Gao et al., 2019). An *in vivo* experiment using ethanol-fed mice proved that ethanol could enhance the antibody-induced CD4^+^ T cell immunosuppression and thus promote tumorigenesis (Hunt et al., 2000). A chronic alcohol treatment was identified to accelerate the immunosenescence process of CD8^+^ T cells of rhesus macaques (Katz et al., 2015). Besides, an *in vitro* experiment showed that alcohol consumption could inhibit the T cell proliferation rate compared with water consumption, and an increase in some pro-tumor immune groups of cells, such as Treg cells and MDSCs, might also impair the function of T cells (Zhang and Meadows, 2010). The apoptosis of T cells would also be activated by the alcohol treatment *via* downregulation of the vitamin D receptor (Rehman et al., 2013).

### Areca Nuts

It is well known that the habit of chewing Areca nuts has been widely popular in Southeast Asia, and its positive role in the development of oral precancerous lesion and OSCC has been fully accepted as well (Li et al., 2016). Apart from its genotoxicity, the areca nut might also affect the progress of oral malignant transformation *via* immunomodulation (Figure 3). For lymphocytes, the DNA synthesis process was identified to get inhibited long before (Yang et al., 1979). This phenomenon posed the hypothesis that areca nuts might decrease the immunity of lymphocytes. Further study confirmed that T cell activation and IFN-γ production were suppressed by areca nut treatment through induction of oxidative stress (Wang et al., 2007). When it comes to immune cell function, the phagocytosis of neutrophils (Hung et al., 2006), the adhesion and migration of mononuclear leukocytes (Chang et al., 2014), and the differentiation of dendric cells from monocytes (Wang et al., 2012) were all proven to decrease due to areca nuts. In the perspective of inflammatory cytokines, treatment of human immune cells using areca nut extract was identified to increase multiple inflammatory cytokines, such as TNF-α, IL6, IL8, cyclooxygenase-2 (COX2), and prostaglandin E2 (PGE2) (Chang et al., 2009; Chang et al., 2013; Faouzi et al., 2018), as well as decrease the level of IL2 production by spleen cells (Selvan et al., 1991). As for oral keratinocytes, production of PGE2, Prostaglandin I2 (PGI2), IL-6, and TNF-a was also enhanced due to areca components (Jeng et al., 2000; Jeng et al., 2003). Expressions of IL-2 and IL-2 receptor by CD8^+^ cytotoxic T lymphocytes (CTLs) and tumor-infiltrating lymphocytes (TILs) were also reduced under the influence of PGE2, while PGE2 induced CD4^+^ Th2 cell activation (Wustrow and Mahnke, 1996; Li et al., 2013). As mentioned above, TILs are lymphocytes that migrate from the blood to the tumor, playing crucial roles in either pro-tumorigenesis or anti-tumorigenesis. Among them, CD4^+^Th2 cells promote tumor growth, while CTLs inhibit tumorigenesis (Lauerova et al., 2002; Farhood et al., 2019). In other words, PGE2 serves as an immunosuppressor contributing to the induction of CD4^+^ Th2 cells and the pro-tumor efficacy of TILs. Circulating the immune complex, known to exhibit an immunosuppressive effect on NK cells and CTLs, was detected to accumulate more frequently in areca chewers than in healthy controls (Remani et al., 1988). A large-population experiment using flow cytometry and immune-staining reveals that IL-17 was highly expressed in areca chewers (Quan et al., 2020). Exposure of areca extracts was shown to induce the increasing secretion of IL-6 and IL-8 by peripheral blood mononuclear cells (Chang et al., 2006). In an animal model, arecoline receivers exhibited a low splenic lymphocyte proliferation rate and a high apoptosis rate (Dasgupta et al., 2006). Similarly, production of IL-8 from oral squamous cancer cells was also increased due to exposure of arecoline (Cheng et al., 2000). PBMC isolated from areca chewers exhibited a higher level of DNA damage markers in circulating lymphocytes (Liu et al., 2004).

### Periodontal Infection

**FIGURE 4:**
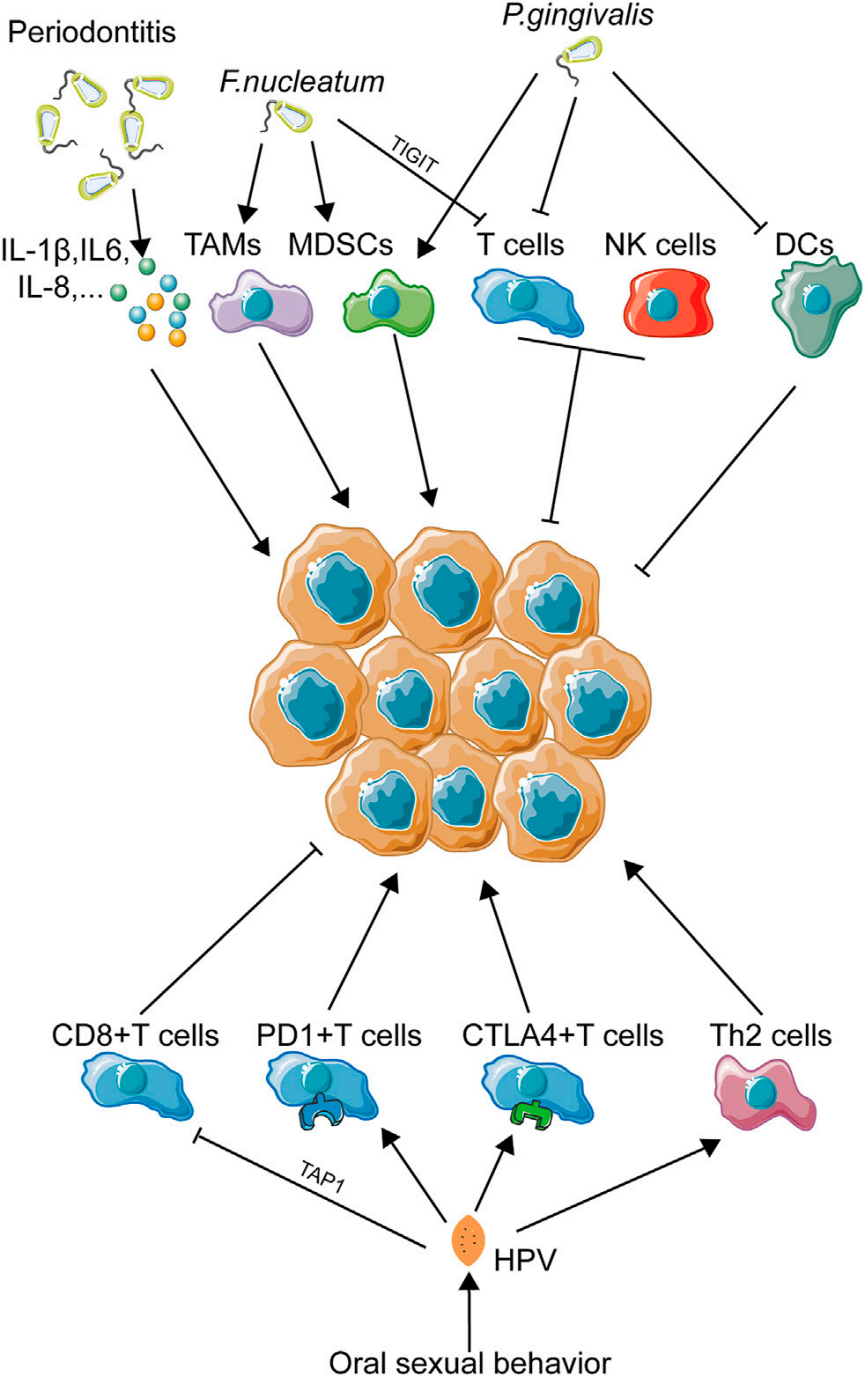
Immune landscape in oral pathogen–related OSCC. This figure depicts the immune landscape caused by oral pathogens, including bacteria and viruses, in OSCC development. Immune regulation was the main activity upon oral pathogen exposure. As infectious factors have been proven to be more and more important in OSCC initiation and progression, immune activities induced by OSCC-related pathogenic bacteria and viruses might also be a crucial part contributing to OSCC progression.

Periodontitis, one of the most common diseases inside the oral cavity, is largely caused by poor oral hygiene status and oral microbial dysbiosis (Lertpimonchai et al., 2017; Meuric et al., 2017). Periodontitis is featured by the dysbiotic inflammatory status (Hajishengallis, 2015), which is highly associated with inflammatory microenvironmental abnormality (Figure 4). Most of pathogenic oral bacteria are Gram negative ones, sharing a similar ability to induce higher concentration of cytokines from oral epithelial cells, such as IL-6, IL-1β, TNF-α, and IL-8 (Ha et al., 2016; Cardoso et al., 2018), and overexpression of these inflammatory cytokines contributes to the abnormality of the microenvironment as discussed above. In particular, some periodontal pathogenic microbiota has been reported to impose immunosuppression on the local focus. *F. nucleatum,* a common periodontitis-associated bacterium, was identified to recruit MDSCs, a kind of tumor-infiltrating immune cell with anti-immunity ability (Kostic et al., 2013). M2 polarization, leading to the differentiation of tumor-associated macrophages, was also observed to be induced by *F. nucleatum* (Chen et al., 2018)*.* TIGIT, a membrane protein of many immune cells, such as NK cells and T cells, could also be modulated by *F. nucleatum,* resulting in loss of function of NK cells and cytotoxic T cells (Gur et al., 2015). Another common periodontal pathogen, *P. gingivalis*, is also involved in immunomodulation. *P. gingivalis* was identified to silence innate immune response partly by inactivating DCs (Abdi et al., 2017). *In vivo* experiment exhibited that *P. gingivalis* infection promoted the expansion of MDSCs (Su et al., 2017). Disturbance of the Th1/Th17 balance was also induced by *P. gingivalis* (Monasterio et al., 2019), while suppression of IL-2 accumulation in T cells (Khalaf and Bengtsson, 2012) and reduction of CXCL10 expression caused by *P. gingivalis* infection (Jauregui et al., 2013) might be used to partly explain this phenomenon. M2 polarization was also elicited by *P. gingivalis* and could promote carcinogenesis in HNSCC (Utispan et al., 2018).

### Oral Sexual Behavior

With the development of society and subsequent changes in traditional concepts, the frequency of oral sexual behavior has mounted to a high level, especially in young adults (Holway and Hernandez, 2018). This behavior shift makes the oral cavity exposed to a totally new environment. Clinical trials have revealed that changes in sexual behaviors trend toward a higher incidence of oral HPV infection (Chaturvedi et al., 2015). This phenomenon just coincides with the conclusion that the percentage of HPV-positive oropharyngeal carcinomas has risen from 16.3% in the 1980s to 72.7% in the 2000s (Chow, 2020). Obviously, oral sex–mediated HPV exposure has become a newly emerging risk factor for oral and pharyngeal carcinomas. It is worth noting that HPV infection has been identified to play a crucial role in local immune disruption (Figure 4). Studies have revealed that HPV^+^ HNSCC patients are more likely to exhibit an abnormal tumor immune microenvironment (Gameiro et al., 2018). A meta-analysis concludes that dysfunction of T cells plays a great part in HPV-induced immune deficiency, while the abnormality of macrophages, Tregs, and MDSCs remains unclear (Lechien et al., 2019). Viral protein E7 could reduce expression of TAP1, as a way to inactivate cytotoxic T cells (Einstein et al., 2009). Infection of HPV would result in downregulation of pro-inflammatory cytokines and upregulation of anti-inflammatory cytokines, such as IL-10 (Mota et al., 1999). In cervical cancer, another HPV-associated carcinoma, loss of T cell cytotoxicity, increase in immunosuppressive Th cell infiltrating, and secretion of immunosuppressive cytokines are all associated with HPV infection (Piersma, 2011). In HNSCC, a large-population transcriptome analysis revealed a T cell dysfunction and T cell exhaustion signatures in HPV-positive patients (Krishna et al., 2018). Furthermore, overexpression of PD-1 and CTLA-4 was observed in HPV-positive HNSCC tissues, which indicated a loss-of-function status of CD8^+^ T cells due to HPV infection (Kansy et al., 2017). In short, abnormality of the T cell status induced by HPV infection might be closely related to OSCC development.

## Risk Factor–Based Immunotherapeutic Strategy

**FIGURE 5:**
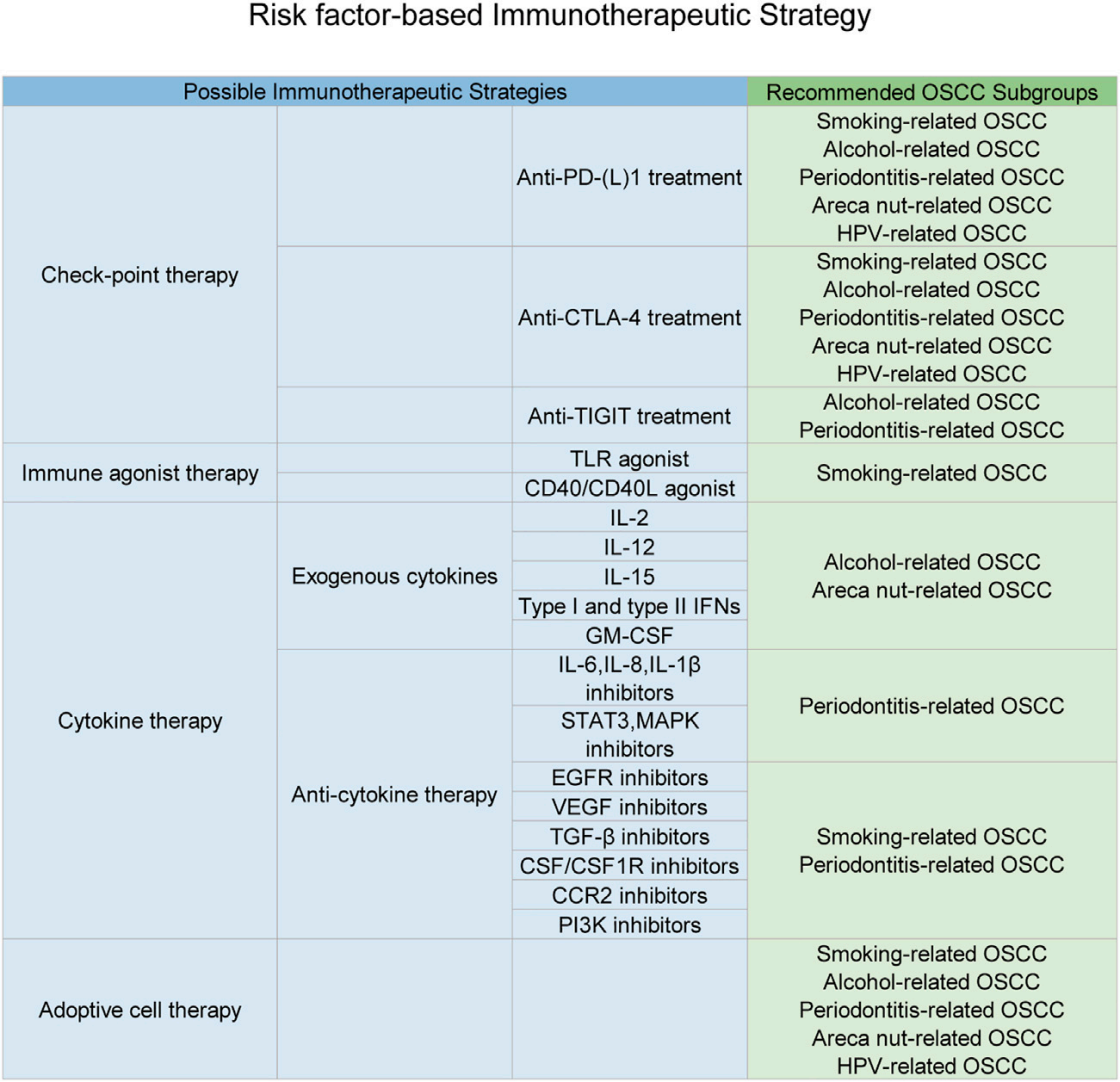
Immunotherapeutic strategies based on the specific carcinogen-related immune status. This figure depicts multiple types of immunotherapeutic strategies and subsequent OSCC subgroups suitable for each kind of strategy. As discussed in this manuscript, carcinogens would result in a specific aberrant immune status for OSCC patients, which just explained drug resistance and individual variations of immunotherapeutic responses. Thus, possible personalized immunotherapeutic strategies based on different carcinogen-induced types of the OSCC local immune status were listed above and might achieve a better clinical outcomes.

Until now, surgical operation is still the first choice for OSCC treatment. Heterogeneity acts as one of the main traits in head and neck squamous cell carcinoma (HNSCC) (Schubert et al., 2020), and poor clinical outcomes of radiation and chemical treatment were partly due to the heterogeneity of OSCC patients (Kagohara et al., 2020). Immune phenotypes of HNSCC classified by the heterogeneity of immune landscapes among HNSCC patients have been built up successfully (Feng et al., 2020). This result further indicated that immune heterogeneity of HNSCC might be summarized into a statistical rule, which might be used for classification of HNSCC patients with different immune statuses. A recent study has identified that smoking could exert an immunosuppressive effect on the HNSCC tumor microenvironment with the help of multi-omics analysis (de la Iglesia et al., 2020). This discovery just coincides with the above hypothesis that oral cancer–related risk factors might greatly account for the abnormality of the immune status. In the perspective of tumor therapy, immunotherapeutic strategies for OSCC ought to be dependent on intratumor heterogeneity to achieve better clinical outcomes (Mroz et al., 2020). Thus, in consideration of the correlation between risk factors and immune variations, a new concept is brought out that combination of patient risk factor information and immune status detection might be valuable for directing individual-based immunotherapy for OSCC patients (Figure 5).

### Check Point Blockade Therapy

#### Anti–PD-(L)1 Treatment

Programmed death ligand 1 (PD-L1) is often expressed on the surface of antigen-presenting cells (APCs), tumor cells, etc., and it can bind to PD-1 on the surface of activated T cells, leading to the exhausted status of T cells (Goodman et al., 2017). Anti–PD-(L)1 treatment could be applied due to its role in reducing T cell apoptosis and enhancing recruitment of T effector cells to tumor sites (Dong et al., 2002). Downregulation of T cell function is a significant feature in OSCC, which is a plausible reason for possible application of anti–PD-(L)1 treatment in OSCC. As reported, anti–PD-1 (aPD1) immunotherapy has been proven to be effective in lymphomas (Goodman et al., 2017), melanoma (Wang et al., 2016), and non–small-cell lung cancer (Xia et al., 2019). Increased expression of PD-1 and PD-L1 was observed in oral lesions progressing to OSCC compared to non-progressing dysplasia (Dave et al., 2020). In addition, several reports revealed that recurrent/metastatic HNSCC patients treated with anti-PD1 showed a significantly prolonged survival compared with standard treatment (Ghanizada et al., 2019). Additionally, high expression of PD-1 was observed in exhausted NK cells, and anti-PD1 therapy could reverse this condition in many cancers (Romero, 2016; Li et al., 2018). So the anti–PD-(L)1 method might also be used as the NK cell–targeted method in alcohol-related OSCC. Subsequent clinical experiments identified that anti-PD1 therapy achieved a better prognostic outcome in HPV^+^ cancer patients than HPV^-^ controls (Ferris et al., 2016). Besides, a systemic meta-analysis confirmed that HPV^+^ HNSCC patients could benefit more from anti-PD1 immunotherapy, further ensuring the role of the PD1 blocking method in the treatment of HPV^+^ OSCC patients (Galvis et al., 2020). In conclusion, anti–PD-(L)1 treatment is an optional method for all subgroups of OSCC discussed above.

#### Anti–CTLA-4 Treatment

The cytotoxic T-lymphocyte–associated antigen 4 (CTLA-4) is a critical receptor for the negative regulation of T cell activation (Rowshanravan et al., 2018). Although elimination of CTLA-4 can result in several diseases including autoimmune diseases, effective anti-tumor immunity sometimes requires the blockade of CTLA-4 (Brunner-Weinzierl and Rudd, 2018; Hosseini et al., 2020).

Wang et al. (2019) found that syngeneic animal models of tobacco-associated oral cancers have higher response rates to anti–CTLA-4 immunotherapy than to anti-PD1 treatment. Anti–CTLA-4 treatment could also increase IFN-γ–producing CD4^+^ Th1 cells, which is necessary for overcoming the imbalance of Th1/Th2 caused by smoking (Chen et al., 2009). On the other hand, downregulation of T cell function was also observed in alcohol-drinking patients. Depending on this point, anti–PD-(L)1 and anti-CTLA4, two common check-point inhibitors for T cells, could be used as discussed above. In addition, based on the overexpression of CTLA-4 observed in HPV^+^ HNSCC tissue samples, a combination of anti-PD1 and anti–CTLA-4 therapies might be likely to achieve a better clinical outcome in HPV-related OSCC patients.

#### Anti-TIGIT Treatment

The T cell immunoreceptor with immunoglobulin and ITIM domain (TIGIT) is a promising new target along with PD-(L)1 and CTLA-4 for cancer immunotherapy, and the blockade of TIGIT and PD-L1 were found to act synergistically on T cells and NK cells’ effector functions (Johnston et al., 2014). Zhang et al. (2018) found that the inhibition of TIGIT could prevent NK cell exhaustion and promote NK cell–dependent tumor immunity in several tumor mouse models. Since the dysfunction of NK cells plays an important pathogenic role in drinking patients, anti-TIGIT therapy might be effective in the treatment of alcohol-related OSCC. In addition, due to the binding ability of *F. nucleatum* to TIGIT and subsequent downregulation of NK cells and T cells, some anti-TIGIT antibodies, MK-7684, for example, might work against the tumorigenesis effect of *F. nucleatum* specifically. As anti-tumor therapies targeting TIGIT have achieved great success recently, this method might also be useful in OSCC patients with high *F. nucleatum* abundance (Solomon and Garrido-Laguna, 2018).

### Immune Agonist Therapies

Although many patients have benefited from checkpoint-blockade immunotherapies, and the overall survival of patients was significantly prolonged due to these therapies, substantial patients do not respond to these strategies, and several drug-resistance mechanisms have been identified (Dempke et al., 2017). To overcome low efficiency of checkpoint-blockade immunotherapies for some OSCC patients, more and more investigations begin to focus on co-stimulatory agents. Toll-like-receptor (TLR) agonists could promote innate immune cells (e.g., macrophages and plasmacytoid DCs), while PD-1 inhibitors act on adoptive immune cells (e.g., activated T cells). Sato-Kaneko et al. (2017) identified that combination therapy with TLR agonists and anti–PD-1 increased antigen-presenting functions of TAMs and the infiltration of IFNγ+CD8+T cells in head and neck tumors, thus suppressing tumor growth. CD40 is a TNF receptor superfamily member expressed on both immune and non-immune cells, and CD40/CD40L agonists could upregulate antigen presentation machinery, enhance T cell proliferation and cytokine production, thus acting in the regression of tumors (Bennett et al., 1998; Mayes et al., 2018; Vonderheide, 2020). Considering the contribution of CS-induced dysfunction of DCs to the development of smoking-related OSCC, these immunotherapies might be effective strategies targeted at these subgroups.

### Cytokine Therapy

#### Exogenous Cytokines

IL-2 was the first approved cytokine for boosting NK cells clinically (Floros and Tarhini, 2015) and was mainly used to produce lymphokine-activated NK cells. Similarly, IL-15 and IL-12 were also pro-inflammatory cytokines playing important roles in the development, homeostasis, and cytotoxicity of NK cells (Floros and Tarhini, 2015). For OSCC patients with a drinking habit, a great burden on NK cells was a significant feature, and these cytokines were the most commonly applied cytokines for NK cell activation, clinically. Apart from their roles in the maturation of NK cells, IL-15 and IL-12 can also lead to IFNγ production (Berraondo et al., 2019). Both type I and type II IFNs have been reported to induce the anti-tumor activities of almost all immune cells, especially the maturation of DCs for antigen presentation and the negative regulation of MDSCs (Parker et al., 2016). Besides, oncolytic virus talimogene laherperepvec (T-VEC), which could express myeloid cell growth and survival factor GM-CSF (Andtbacka et al., 2019), might also be used to ameliorate the loss of DCs in smoking OSCC patients. Considering that inactivation of DCs and induction of MDSCs were both related to high abundance of *P. gingivalis* and areca-chewing habits, these cytokines mentioned above might be effective for those OSCC patients. In addition, owing to the significant decrease in IL-2 induced by areca-chewing, an extraordinary supplementation of IL-2 might also help to some extent.

#### Anti-Cytokine Therapy

Anti-cytokine therapy here refers to blockades of cytokines, cytokine receptors, and the subsequent signaling pathways. Overexpression of some crucial pro-inflammatory cytokines, including IL-6, IL-8, and IL-1β, was a shared phenomenon during periodontitis. These cytokines were reported to promote tumorigenesis (Kumari et al., 2016; Berraondo et al., 2019), so monoclonal antibody therapy against them or their signaling downstream molecules such as STAT3 and MAPK might help a lot (Johnson et al., 2018).

Tumor-associated macrophages (TAMs), which contribute to local tumor growth, often express some angiogenesis-promoting factors (e.g., EGFR ligands and VEGF) and immune-suppressing factors (e.g., TGF-β and IL-10), contributing to tumor growth and metastasis. Thus, monoclonal antibody therapies targeting these molecules could relieve the tumor burden to some extent. Inhibition of CSF1/CSF1R could suppress proliferation, differentiation, and survival of monocytes and macrophages and has been examined through multiple types of cancers (Strachan et al., 2013; Chitu and Stanley, 2017). In addition, CCR2 inhibition and PI3K inhibition, which could be used to restrict TAM recruitment into tumor sites (Okkenhaug, 2013; Le et al., 2018), might also reduce the recruitment and accumulation of TAMs into OSCC local lesions. Since TAMs tend to get accumulated inside tumor sites in OSCC patients with a smoking habit or high abundance of *F. nucleatum*, the strategies targeting TAMs discussed above were necessary.

### Adoptive Cell Therapy

Adoptive cell therapy (ACT) is a new form of immunotherapy in which autologous immune cells (mainly T cells) from peripheral blood were engineered *ex vivo* to express tumor-specific transgenic antigen receptors such as chimeric antigen receptors (CARs) or T cell receptors (TCRs) (Wang and Cao, 2020). Despite the application of CD19-directed CAR-T cells having shown remarkable success in the treatment of CD19^+^ B cell malignancies, there are some obstacles to this method for solid tumors due to the heterogeneity of antigens expressed in solid tumors (Chan et al., 2021) and the immunosuppressive tumor microenvironment (Yeku et al., 2017). However, investigations into its solutions never stop. CAR-T cell therapy targeting ErbB family receptors has attracted a lot of interest in the treatment of head and neck cancer and was evaluated in ongoing phase I clinical trails (van Schalkwyk et al., 2013; Yeku et al., 2017). In addition, engineered T cells expressing the dendritic cell growth factor Flt3L were reported to overcome the clinical problem of antigen-negative tumor escape following ACT (Lai et al., 2020). Besides, many scholars demonstrate that the combination of ACT and approaches targeting immune check-point receptors would enhance anti-tumor immunity *in vivo* (Liu et al., 2017; Chan et al., 2021). Similarly, other therapies, such as NK cell adoptive transferring and NK cell manufacture, which have not been broadly used, might also be applied in clinical treatment in the future. In conclusion, ACT, mentioned above, has a promising future in OSCC treatment and helps solve the T cell and NK cell exhaustion in TME even though more scientific research studies are still necessary.

## Discussion

In this review, an abnormal immune status during the progress of OSCC was depicted first. Complete analysis of immune abnormalities caused by different oral cancer–related carcinogens was then accomplished. Based on different types of immune abnormalities induced by different carcinogens, possible individual immunotherapeutic strategies dependent on carcinogen-induced immune abnormalities were figured out as a way to possibly overcome heterogeneity of OSCC patients and enhance clinical efficacy of immunotherapies.

Nowadays, surgical resection still remains the predominant method for treatment of OSCC. However, the heavy financial and physical burden of surgical operation has become an insurmountable hurdle for lots of OSCC patients (Hamoir et al., 2014). In this condition, some alternative treatment methods, including immunotherapy, deserve further attempts during the treatment process of OSCC. Heterogeneity among different cancer patients acts as a main obstacle for common applications of immunotherapies among multiple cancers, OSCC included (Caswell and Swanton, 2017). Until now, multiple studies have focused on creating personalized therapies for HNSCC (Baird et al., 2018), and subgroup division seems to be an effective way for this. Until now, some subgroup division standards based on HPV status, age, and cetuximab history have failed to contribute greatly to enhancing the efficacy of immunotherapy in OSCC (Cramer et al., 2019). Different types of local immune abnormalities caused by different carcinogens suggest that subgroup division standard based on carcinogens, in combination with specific judgment of the patient immune status, might help achieve individual-targeted immunotherapy and improve the clinical outcome of immunotherapy-treated patients to a great extent.

Nevertheless, lack of systemic studies for influence of different carcinogens on the OSCC local immune status is a defect for our review, so we could only predict possible immune alternations and corresponding therapeutic strategies for OSCC. Further studies are still necessary to verify these possibilities. Besides, occurrence of OSCC depends not only on carcinogens but also genetic abnormalities, which means carcinogen-based immunotherapies might not completely explain and overcome heterogeneity of OSCC patients. In addition, a specific OSCC patient might be under the influence of more than one type of carcinogen, which might render the local immune microenvironment more complex and harder to be predicted. Multiple factor–associated OSCC has long become a challenge for chemotherapy, as different types of carcinogens would produce a complex network regulating patterns, making it an extreme dilemma for researchers. Until now, studies about OSCC chemotherapy have all focused on the influence of a single carcinogenic factor, while no such clinical study aimed at figuring out multiple factor–induced immune variations has been completed yet. Based on this research status, efforts were made in this manuscript to describe and summarize immune status variations induced by every specific OSCC carcinogen and changes of a specific immune status associated with multiple types of OSCC carcinogens. Thus, our review only aimed at a complete analysis and summarization of current knowledge about single-carcinogen–induced immune abnormalities in OSCC. Obviously, more clinical experiments focused on this issue ought to be conducted to confirm our assumption, constructing a better strategy to expand application of immunotherapies during the OSCC treatment process.
